# Editorial for the Special Issue on Piezoelectric Nanogenerators for Micro-Energy and Self-Powered Sensors

**DOI:** 10.3390/mi13091443

**Published:** 2022-09-01

**Authors:** Micky Rakotondrabe, Rusen Yang, Zhong Lin Wang

**Affiliations:** 1 Laboratoire Génie de Production, National School of Engineering in Tarbes (ENIT), National Polytechnic Institute of Toulouse (INPT), University of Toulouse, 65000 Tarbes, France; 2 Academy of Advanced Interdisciplinary Research, School of Advanced Materials and Nanotechnology, Xidian University, Xi’an 710126, China; 3 School of Material Science and Engineering, Georgia Institute of Technology, Atlanta, GA 30332, USA

Energy harvesting consists of scavenging energy from the surrounding environment knowing that this energy would be “lost” if not scavenged. To scavenge small-scale kinetic energy, the use of a piezoelectric nanogenerator (PENG) is one of the most studied and developed approaches. It is based on the exploitation of the direct piezoelectric effect to transform the ambient kinetic energy—mostly vibrational—into electrical energy. While the term PENG was initially introduced when referring to ZnO nanowires being used as materials to scavenge such small-scale energy, the word nowadays refers to piezoelectric energy harvesting more generally, whether standard materials (e.g., PZT, AlN) or novel materials (e.g., nanowires) are employed. Indeed, the driving mechanism of PENG is Maxwell’s displacement current. Potential applications of PENG are numerous, as it allows self-powered and autonomous nano-, micro-, mini-, and meso-scale devices, for example, implantable electronics in biomedical applications, geotracers and animal tracking devices, wearable devices, multifunctional shoes, tires monitoring sensors, autonomous sensors in automotives, building monitoring sensors, and self-powered vibration damping devices in structures. Nowadays, we are witnessing a variety of attractive approaches in the emerging research and development of increasingly more efficient PENGs with more diversified applications. This Special Issue aims to present a collection of articles, including a review paper, that cover the recent research and development on PENG techniques and their applications, as well as on piezoelectric energy harvesting. Collectively, the papers in this issue address fundamental, technological, and application aspects.

After a high-quality reviewing process, ten papers have been accepted to be published in this Special Issue. Each of them is quickly introduced below.

In [1], Kumari and Rakotondrabe present a design for harvesting small-scale kinetic energy using a structure based on lithium niobate (LiNbO3) piezoelectric material and multiple cantilevers. The material used is lead-free, and the structure allows energy to be harvested from wideband, low-frequency, and low-amplitude vibrations. The article presents both a theoretical aspect and fabrication and an experimental part.

The second article referenced in [2] reports the effect of dielectric constant on electrical outputs of a piezoelectric nanogenerator that uses ZnO/PDMS composites with varied ZnO coverages. The authors, Amangeldinova et al., adjusted the dielectric constant of the piezoelectric layers from 3.37 to 6.75. Overall, the authors concluded that the output voltage of the piezoelectric nanogenerator comprising the piezoelectric composite with the lowest dielectric constant was found to be boosted approximately 11 times compared to that of the nanogenerator with the highest dielectric constant. The verification was made with simulation and experiments.

In [3], Zhang et al. show interest in energy harvesting systems based on piezoelectric ceramics materials (piezoceramics). Considering the piezoceramics as a current source, the authors develop a current output model to accurately estimate its value under various kinds of stimulation such as with diverse frequency, diverse amplitude, or diverse preload. The article presents both simulation and experimental results.

The next paper [4] is by Amiri et al. The authors provide a full analysis of the specific case of piezoelectric nanostructures that are semiconductive and under axial strain, with applications including energy harvesting. For this purpose, an example of nanostructure is taken: a nanostructure that has a conic shape, based on ZnO piezoelectric material, and is under axial force. After providing the definitions of some parameters related to piezopotential, the authors performed the analysis using COMSOl Multiphysics finite-element-method software and quantitatively evaluated the parameters when the axial force is applied.

In [5], Zuo et al. developed fluorinated polyethylene propylene (FEP) bipolar ferroelectret films with a concentric tunnel structure. Then, they characterized the properties, including piezoelectric-thermal and mechanical, of the fabricated films for possible two kinds of energy harvesting: working in 33 and in 31 modes. The authors show experimentally that the FEP films exhibit significant longitudinal and radial piezoelectric activities, as well as interesting thermal stability.

In [6], Xu et al. specifically studied the piezoelectric circular diaphragm (PCD)-based energy harvester. The authors found that the strain distribution in such a PCD is non-uniform, as is the voltage distribution. On the basis of this observed phenomenon, the authors proposed an optimized version of the PCD structure in order to increase the output power, which is afterwards verified experimentally.

Elahi et al. present in [7] an energy harvester based on a flag-flutter mechanism and using lead-zirconate-titanate (PZT) piezoelectric material. The harvesting principle uses fluid–structure interaction, and for this purpose, the mechanism is subjected to the axial airflow in a subsonic wind tunnel. The authors consider different configurations of the flag and analyze them through simulation and experiments.

In [8], Elvira-Hernandez et al. present an analytical electromechanical modeling of a piezoelectric nanogenerator composed of a double-clamped beam with five multilayered cross-sections. The structure is composed of a central seismic mass, substrate of polyethylene terephthalate (PET), and ZnO piezoelectric material with aluminum electrodes. The simulation results of the analytical model are afterward compared with finite-element-method (FEM) simulation results using ANSYS software, which show agreement between them.

Hommayouni-Amlashi et al. present in [9] the optimization of piezoelectric cantilever energy harvester with an in-span attachment. By using a neural-network-based genetic algorithm (NN-GA), the authors found the attachment masses, attachment mass moment of inertia, their location, the piezoelectric patches locations, and the applied force location along the cantilever, in order to maximize output voltage for a given excitation frequency. The optimization was validated with COMSOL Multiphysics finite-element-method software. The authors mention that the advantage of the optimization approach is that it can handle many more parameters than other techniques such as topology optimization [10], interval techniques [11], or model-simulation-based optimization [12].

The last paper is by Han et al. [13] and is a review of a triboelectric nanogenerator (TENG) and on piezoelectric transducer (PE)-based self-powered sensors for robotic applications. The authors recall the principles of TENG and PE first. Afterwards, they give the research status on pressure sensors, displacement sensors, and three-dimensional (3D) acceleration sensors based on TENG and on PE, separately, and then on a PE-TENG hybrid principle.
